# Profiles of Microbial Community and Antibiotic Resistome in Wild Tick Species

**DOI:** 10.1128/msystems.00037-22

**Published:** 2022-08-01

**Authors:** Nana Wei, Jinmiao Lu, Yi Dong, Shibo Li

**Affiliations:** a Department of Infectious Disease, Zhoushan Hospital, Wenzhou Medical University, Zhoushan, China; b Key Laboratory of Adolescent Health Assessment and Exercise Intervention of Ministry of Education, East China Normal University, Shanghai, China; c Guangdong Provincial Zoonosis Prevention and Control Key Laboratory, College of Veterinary Medicine, South China Agricultural University, Guangzhou, China; University of California San Diego

**Keywords:** wild ticks, microbiome, antibiotic resistance, antibiotic resistance genes (ARGs), tick, microbiome

## Abstract

Infections caused by antibiotic-resistant pathogens pose high risks to human and animal health worldwide. In recent years, the environment and wildlife as major sources and reservoirs of antibiotic resistance genes (ARGs) are being increasingly investigated. There have been many reports on bacterial community in ticks, but little is known about ARGs they carry, and the correlation between bacterial and ARGs in wild ticks also remains unknown. Here, the profiles of microbial community and antibiotic resistome in wild tick species were investigated using high-throughput 16S rRNA sequencing and smart chip-based high-throughput quantitative PCR approach (HT-qPCR), respectively. We found that bacterial composition in wild tick species is variable; the sequenced reads from all samples were assigned to 37 different phyla at the phylum level. The dominant phylum was *Proteobacteria*, which accounted for 75.60 ± 10.34%, followed by *Bacteroidetes* accounting for 13.78 ± 11.68% of the total bacterial community. In total, 100 different ARGs across 12 antibiotic classes and 20 mobile genetic elements (MGEs) were identified by HT-qPCR, and among them aminoglycosides, multidrug, macrolide-clinolamide-streptogramin B, and tetracycline resistance genes were the dominant ARG types. Co-occurrence patterns revealed by network analysis showed that eight bacterial genera may serve as the potential hosts for different ARGs. For the first time, this study provides comprehensive overview of the diversity and abundance of ARGs in wild ticks and highlights the possible role of wild ticks as ARG disseminators into the environment and vertebrate hosts, with implications for human and animal health.

**IMPORTANCE** The emergence of antibiotic-resistant bacteria poses serious threat to the public health around the world. Ticks are obligate hematophagous ectoparasites, surviving via feeding on the blood of various animal hosts. Although some previous studies have confirmed wild ticks carried various bacterial community, the role of wild ticks in the antibiotic resistance remains unknown. Here, identification of microbial community and antibiotic resistome in wild tick species revealed that wild ticks are the reservoir, postulated potential spreaders of antibiotic resistance. Our findings highlight the contribution of wild ticks to the maintenance and dissemination of ARGs, and the associated health risks.

## INTRODUCTION

Antimicrobial resistance (AMR) is of major concern as one of the global public health problems of the 21st century, which seriously threatens many of the most important medical advances we have made (1). In recent years, widespread use and abuse of antibiotics in human, veterinary medicine, and agricultural arenas have led to sharply increased morbidity and mortality, and considerable global economic cost. Without decisively action to tackle the current trend of rising AMR, it is estimated that AMR infections would cause 10 million deaths by 2050 and a reduction of 2% to 3.5% in Gross Domestic Product (2). Antibiotic resistance is a One Health challenge—the health of people is connected to the health of animals and the environment (3). AMR is not just a regional or national problem; AMR as a global multifaceted phenomenon has been widely studied in the microbiome of human, livestock, and migratory birds (4–6). Studies have demonstrated that both poultry and avian gut microbiomes contains high diversity and abundance of antibiotic resistance genes (ARGs) (6–8). Hu et al. (9) identified a total of 1,093 ARGs from 162 individuals gut microbiota, and Xiao et al. (10) reported the common profile of ARGs found in all pigs. Nevertheless, little is known about the characteristics and distributions of ARGs of the bacterial species of the wild tick microbiome, in contrast to the extensive studies of human and other mammals’ microbiome and resistome.

Ticks are exclusively hematophagous ectoparasites responsible for transmitting multiple pathogens, which severely endanger the health and life of both humans and animals (11). Ticks and tick-borne diseases (TBDs) are a global issue due to medical and veterinary public health importance (12, 13). The microbiota is a collection community of microorganisms including bacteria, viruses, eukaryotes, and archaea, which are essential in maintaining host homoeostasis and health. Moreover, a growing body of researches suggest that the microbiota can serve as reservoir of antibiotic-resistant bacteria (ARBs) and their associated ARGs (7, 10). Studies have demonstrated that tick harbor a complex and diverse microbiota. In addition, tick microbiota composition is dependent on environmental and vertebrate hosts (14, 15). Nowadays, ticks are becoming increasingly global due to climate change, migratory birds flight route change and growing imported livestock (16). In addition, urban greening and ecotourism and adventure travel increase the risks for humans and animals of exposure to ticks. Widely geographical distribution and host diversity of ticks make them particularly likely to transmission ARBs and ARGs. Although scientists revealed that ticks might transfer DNA between snakes and lizards and into ruminants and marsupials and could acquire the TAM gene through the horizontal gene transfer (HGT) from vertebrates (17, 18), we know little about the distribution and functional repertoires of tick microbiome genes. We therefore hypothesized that wild ticks may be a reservoir of ARGs and might contribute to the spread of ARGs to the environment and vertebrate hosts. Considering HGT, ARGs present in wild ticks may directly spread to humans via tick biting or via contaminated meat that is from animals bitten by ticks. It is important to figure out the ARG profile and relationship between microbiome and ARGs in ticks for mitigating the dissemination of such vector-borne ARGs.

Recently, rapid functional high-throughput metagenomics have been used to analyze and describe ARGs of human and environmental microbiota (9, 19, 20). Here, to test our hypothesis, we profiled the microbiome and resistome in wild ticks, by applying 16S rRNA amplicon sequencing and HT-qPCR. In the present study, we describe the diversity and abundance of bacterial community in wild ticks collected from different districts in China, depicted the distribution of ARGs within bacterial, and investigated the possible correlation between bacteria and ARGs. For the first time, our findings described the ARG characteristics of bacteria colonized in the wild ticks and showed the potential correlation between the bacterial community and ARGs. These results in our present study suggest that ticks maybe a reservoir of ARGs and may have potential risks to human health and the veterinary industry by transporting ARGs and ARBs.

## RESULTS

### Microbial community composition in wild tick species.

**FIG 1:**
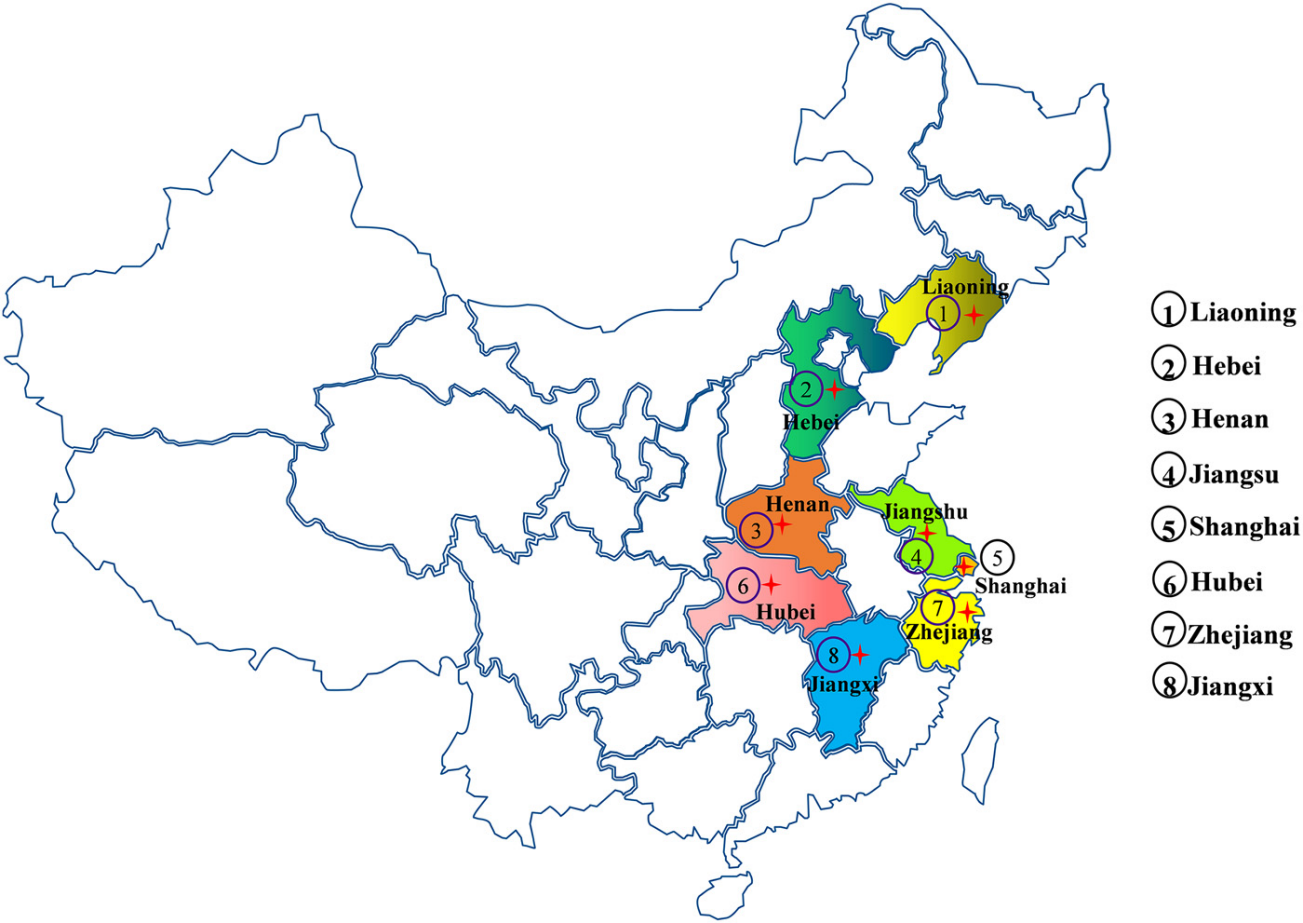
Map showing sampling sites of eight districts in China.

**FIG 2:**
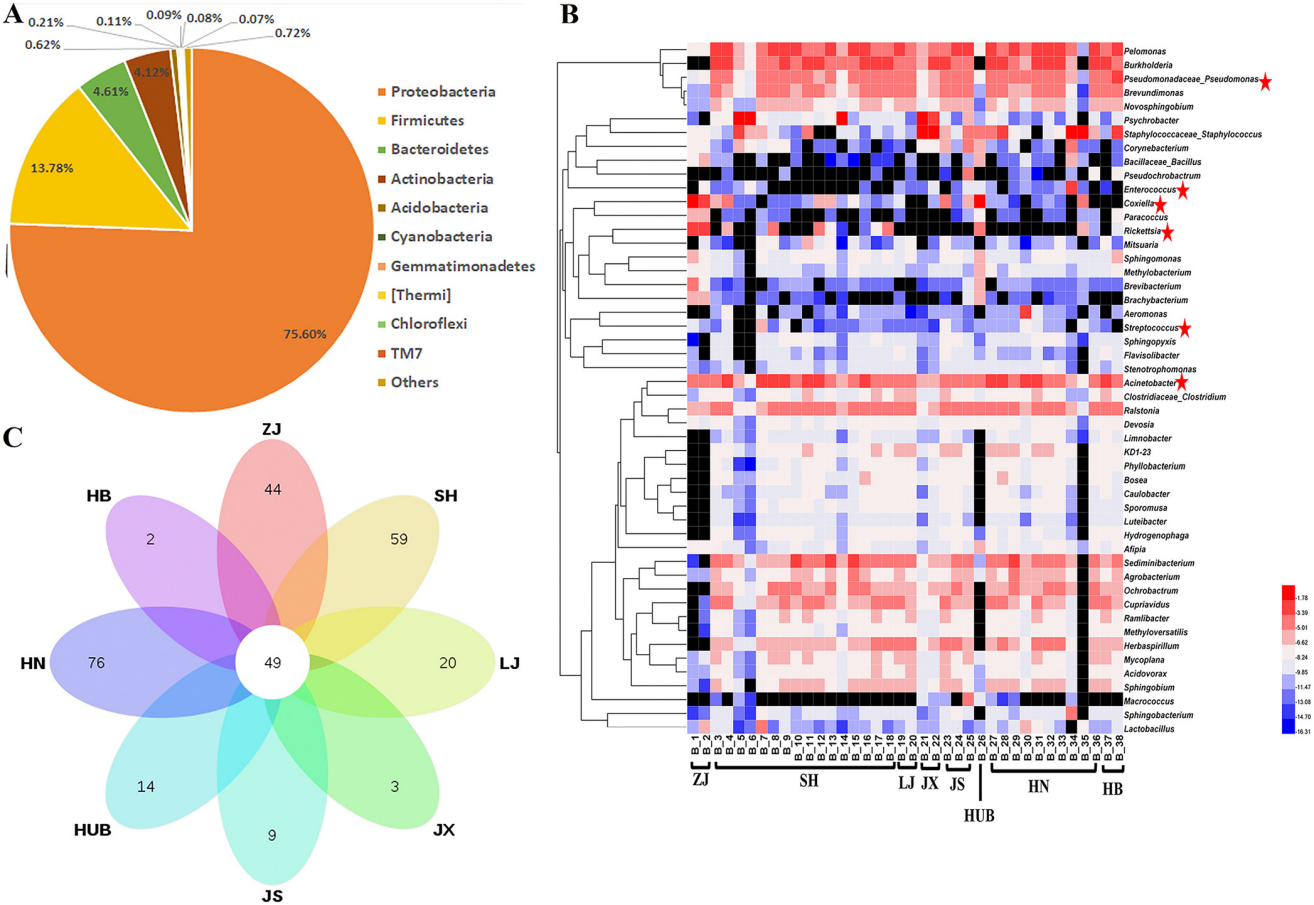
The microbial community composition of the wild tick species from eight different districts in China. (A) Characterization of the bacterial community in the wild tick species at phylum level. (B) The heatmap showing the top 50 genus in each tick pool. (C) Venn diagram showing the shared and unique genera among different wild tick pools. ZJ, Zhejiang; SH, Shanghai; LJ, Liaoning; JX, Jiangxi; JS, Jiangsu; HUB, Hubei; HN, Henan; HB, Hebei. The red asterisk represented the potential human pathogens. Genes not detected are marked in black.

The bacterial community structure of 30-eight tick pools, representing 8 populations collected from the wild across eight different Chinese provinces (Fig. 1) were analyzed using sequence data of 16S rRNA genes. The sequenced reads from all samples were assigned to 37 different phyla at the phylum level. We found that *Proteobacteria* (75.60 ± 10.34%), *Firmicutes* (13.78 ± 11.68%), *Bacteroidetes* (4.61 ± 1.95%), and *Actinobacteria (*4.12 ± 3.34%) were the predominant taxonomic phyla in the microbiota of wild ticks (Fig. 2A). The abundance of *Firmicutes* in some tick pools like B34 and B35 could reach up to 63.91% and 82.59%, respectively (Fig. S1). *Proteobacteria* was dominated by *Gammaproteobacteria*, *Betaproteobacteria*, and *Alphaproteobacteria* (Fig. S2A); *Firmicutes* was largely represented by *Bacilli* and *Clostridia* (Fig. S2B). *Bacteroida*, *Saprospirae*, and *Sphingobacteriia* were the most abundant classes in the phylum *Bacteroidetes* (Fig. S2C); *Actinobacteria*, *iii1-8*, and *Acidimicrobiia* consisted of the class *Actinobacteria* (Fig. S2D).

10.1128/msystems.00037-22.1 FIG S1 Total bacterial abundance at the taxonomic rank of phylum of wild ticks in each tick pool. ZJ, Zhejiang; SH, Shanghai; LJ, Liaoning; JX, Jiangxi; JS, Jiangsu; HUB, Hubei; HN, Henan; HB, Hebei. Download FIG S1, TIF file, 2.7 MB. Copyright © 2022 Wei et al. 2022 Wei et al. https://creativecommons.org/licenses/by/4.0/ This content is distributed under the terms of the Creative Commons Attribution 4.0 International license.

10.1128/msystems.00037-22.2 FIG S2 The classes are presented in four major dominant phyla. (A) Proteobacteria. (B) Firmicutes. (C) Bacteroidetes. (D) Actinobacteria. (E) Venn diagram showing the shared and unique ARGs and MGEs among different wild tick pools. (F) Heatmap showing the occurrence of the top 10 ARG subtypes and MGEs, the numerical colors indicated the values were log2 transformed based on the relative abundance. Download FIG S2, TIF file, 1.7 MB. Copyright © 2022 Wei et al. 2022 Wei et al. https://creativecommons.org/licenses/by/4.0/ This content is distributed under the terms of the Creative Commons Attribution 4.0 International license.

At the genus level, 485 genera were identified across all tick pools, and the dominant genus varied significantly among different tick pools. Overall, *Coxiella*, *Staphylococcaceae*_Staphylococcus, *Pelomonas*, *Burkholderia*, and Acinetobacter are the most abundant genera with the averagely relative abundance of 9.92%, 8.25%, 7.87%, 7.06%, and 6.39%, respectively (Fig. 2B). It is worth noting that a large proportion of the genus *Coxiella* was found in Zhejiang (ZJ) and Hubei (HUB) pools (34.38% and 42.01%, respectively), while the *Rickettsia* was also detected mainly in ZJ and HUB pools (13.23% and 6.85%, respectively). The dominant genera *Pelomonas* and *Burkholderia* were detected in Shanghai (SH), Liaoning (LJ), Henan (HN), and Hebei (HB) pools, while the ticks in Jiangxi (JX) pool harbored the dominant genera *Staphylococcaceae*_Staphylococcus and *Psychrobacter* (37.95% and 30.32%, respectively). A total of 227 district-specific genera were identified, with a relatively low abundance, and 49 genera were shared by all tick pools (Fig. 2C and Table S3). Although 485 genera were identified across all samples, the top 50 most abundant genera represented a large proportion of the microbial community in each tick pool ranging from 71.37% to 94.75% (Table S4).

10.1128/msystems.00037-22.5 TABLE S3 Shared genera and district-specific genera identified in all groups. Download Table S3, XLS file, 0.1 MB. Copyright © 2022 Wei et al. 2022 Wei et al. https://creativecommons.org/licenses/by/4.0/ This content is distributed under the terms of the Creative Commons Attribution 4.0 International license.

10.1128/msystems.00037-22.6 TABLE S4 Top 50 genera in all groups. Download Table S4, XLS file, 0.03 MB. Copyright © 2022 Wei et al. 2022 Wei et al. https://creativecommons.org/licenses/by/4.0/ This content is distributed under the terms of the Creative Commons Attribution 4.0 International license.

A total of six opportunistic pathogens were detected among these samples, and they were prevalent in all the eight tick populations. Some clinically important pathogens were also observed in ticks, such as *Coxiella* and *Rickettsia* (Fig. 2B). The two genera are potential human pathogens, and they have been demonstrated worldwide in ticks; however, all of them contained several nonpathogenic strains as well (21, 22). They also play roles in B vitamins biosynthesis and the transmission of tick-borne pathogens (14). In addition, the genus Acinetobacter has been reported due to its extensive antibiotic resistance spectrum (23), and it was prevalent in all the tick pools in our study (Fig. 2B), which may allow transfer genes to other bacteria making ticks a useful reservoir for pathogens.

### Profiles of antibiotic resistome in wild tick species.

**FIG 3:**
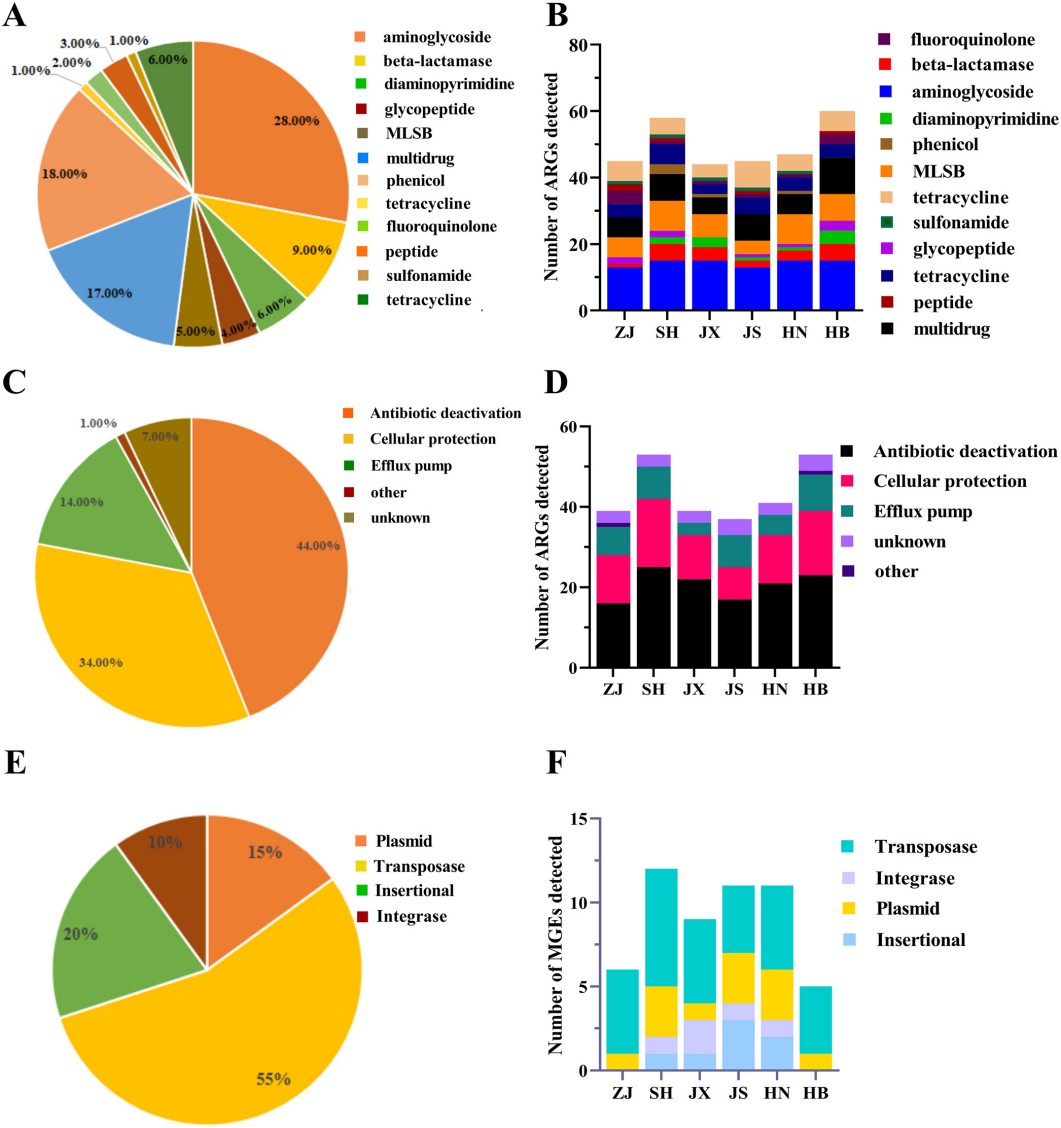
Profiles of resistome of wild tick species from six different districts in China. The percentage (A) and the number (B) of ARGs detected in wild tick species are drawn according to the classes of ARGs. The percentage (C) and the number (D) of ARGs detected in wild tick species are drawn according to the resistance mechanisms. The percentage (E) and the number (F) detected of MGEs in wild tick species are drawn according to the resistance mechanisms.

Six tick pools across six different Chinese provinces were used for the analysis of antibiotic resistance (Fig. 1). Resistance profiles of ARGs were determined through the HT-qPCR method. A total of 384 genes (352 ARGs, 24 MGEs, 7 human pathogens, and the 16S rRNA gene) were detected in the tick samples. A total of 124 genes including 100 ARGs, 20 MGEs, 3 pathogens, and 16S rRNA were detected in the tick samples (Table S5). The dominant ARGs confer resistance to 12 classes of antibiotics, including aminoglycoside (28.00%), beta-lactamase (9.00%), diaminopyrimidine (6.00%), fluoroquinolone (4.00%), glycopeptide (5.00%), macrolide-clinolamide-streptogramin B (MLSB; 17.00%), multidrug (18.00%), peptide (2.00%), phenicol (3.00%), sulfonamide (1.00%), tetracycline (6.00%), and others (1.00%) (Fig. 3A). The total numbers of ARGs detected in each tick pool ranged from 37 to 54, with an average of 44 (Fig. 3B). These ARGs are divided into antibiotic deactivation, cellular protection, efflux pumps, other, and unknown mechanism according to their resistance mechanisms. The dominant resistance mechanisms in our present study were antibiotic deactivation and cellular protection, which accounted for 44.00% and 34.00%, respectively, followed by efflux pumps, which accounted for 14.00% (Fig. 3C and D). Herein, 20 MGEs, including 4 insertional (20.00%), 2 integrase (10.00%), 3 plasmid (15.00%), and 11 transposase genes (55.00%), were detected in the tick samples collected from 6 different districts (Fig. 3E). The main MGEs were transposase among all the tick pools (Fig. 3F). Furthermore, according to the detected genes by HT-qPCR, a total of 41 district-specific genes were identified, and 12 genes that accounted for 10.00% in all detected genes were presented in all these tick pools (Fig. S2E and Table S6).

10.1128/msystems.00037-22.7 TABLE S5 The relative abundance of genes (target gene copies/16S rRNA copies). Download Table S5, XLS file, 0.1 MB. Copyright © 2022 Wei et al. 2022 Wei et al. https://creativecommons.org/licenses/by/4.0/ This content is distributed under the terms of the Creative Commons Attribution 4.0 International license.

10.1128/msystems.00037-22.8 TABLE S6 Shared genes and district-specific genes identified in all groups. Download Table S6, XLS file, 0.02 MB. Copyright © 2022 Wei et al. 2022 Wei et al. https://creativecommons.org/licenses/by/4.0/ This content is distributed under the terms of the Creative Commons Attribution 4.0 International license.

**FIG 4:**
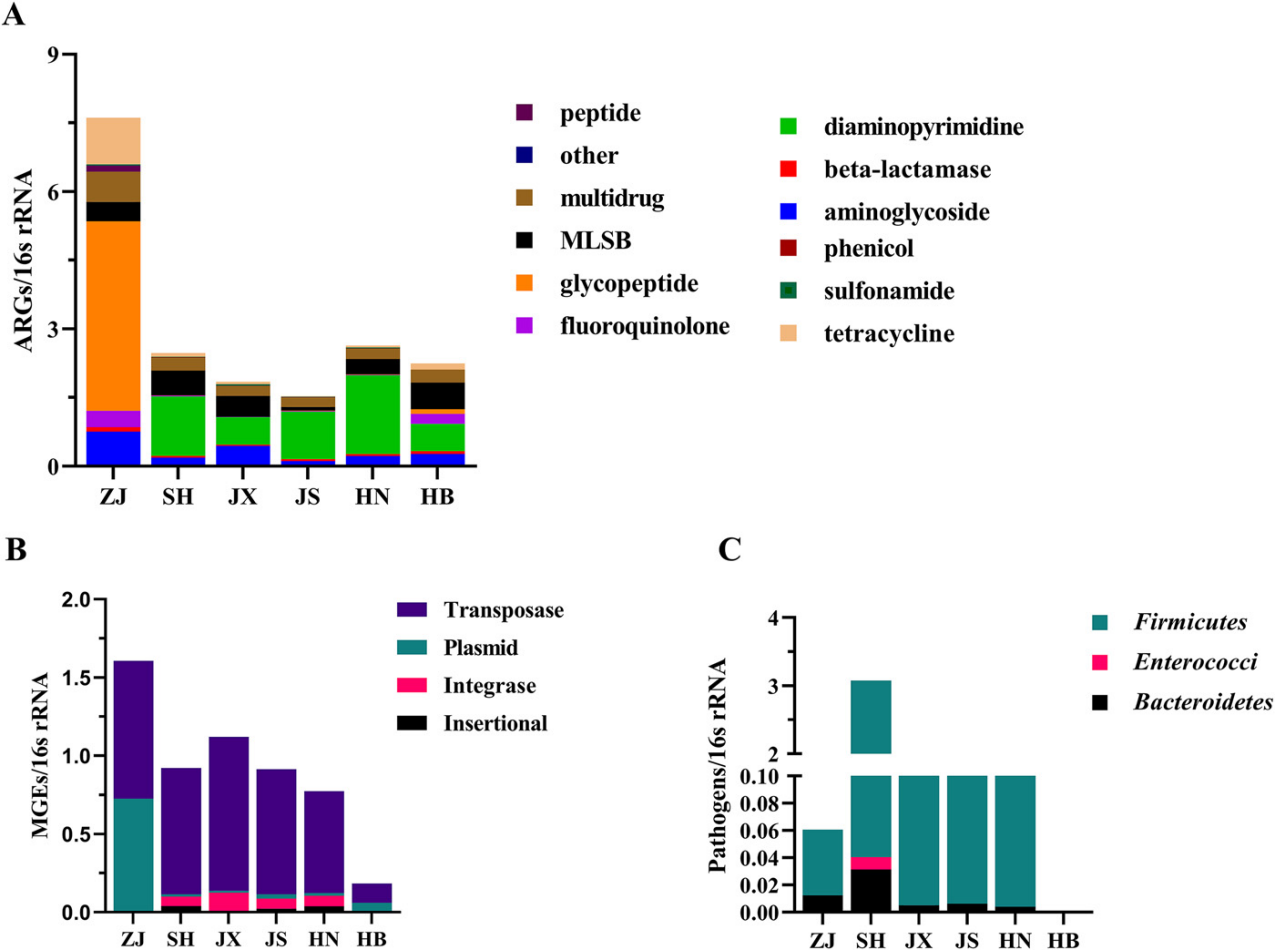
The relative abundance per 16S rRNA gene of ARGs (A), MGEs (B), and pathogens (C) in the wild tick species among different tick pools. Relative abundances of all detected antibiotic resistance genes are provided in Table S6 in supplemental material.

Although there is similar richness of ARGs and MGEs among all the tick pools (Fig. 3), the relative abundance of ARGs in ZJ pool was significantly higher than the other pools (Fig. 4A and B). The glycopeptide resistance genes dominated in ZJ pool, but the diaminopyrimidine resistance genes dominated in the other five pools (Fig. 4A and Fig. S2F). Also, we found that the relative abundance of plasmid in ZJ pool was generally higher than the other pools (Fig. 4B). Both of the relative abundances of MGEs and pathogens in HB pool were the lowest compared to the other tick pools (Fig. 4B and C). The composition and the abundance of ARGs varied among different tick pools, but among the genes found in all tick pools, aminoglycoside, MLSB, and multidrug were consistently detected at a higher abundance (Fig. 4A and Table S5). Our results showed that the abundance, but not the richness of ARGs, in wild ticks may be affected by the environments from different districts, and whether antibiotic usage (method, dose, types) in different districts is the main factor needs further studies.

### Correlations between ARGs and MGEs in wild tick species.

**FIG 5:**
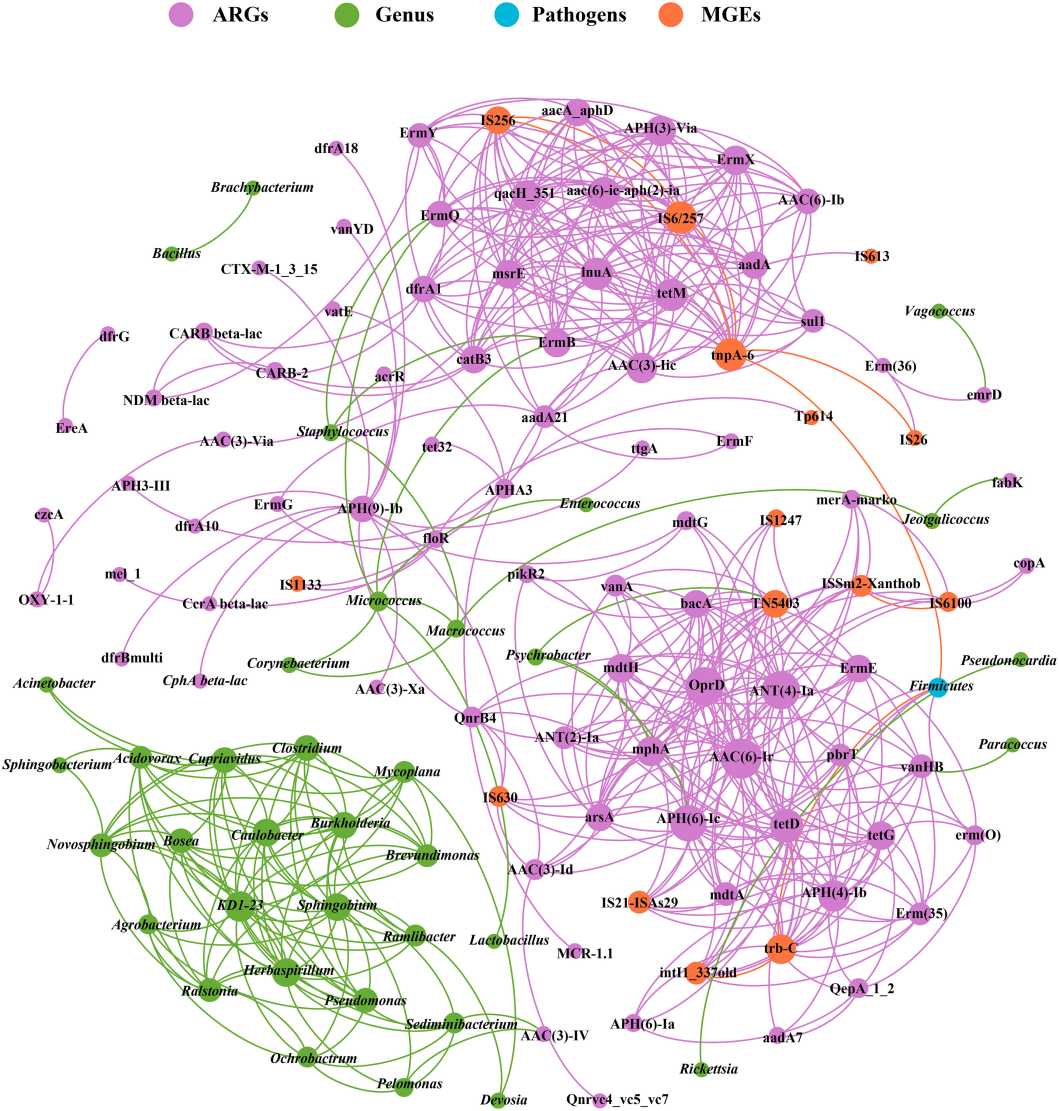
Network analysis about the co-occurrence patterns among ARGs, MGEs and microbial taxa. A connection represents a strong (Spearman’s correlation coefficient *r *> 0.70) and significant (*P < *0.05) correlation. The size of each node is proportional to the number of connections.

A significantly positive correlation between all MGEs and all ARGs existed (*r* = 0.61, *P = *0.01) based on the Spearman correlation analysis. The further correlation analysis indicated that diaminopyrimidine, fluoroquinolone, glycopeptide, peptide, and tetracycline resistance genes were significantly correlated with insertional, integrase, and plasmid, but not transposase (Table S7). To further identify the cooccurrence relationship between ARGs and MGEs, correlation analysis was performed on different types of ARGs and MGEs. For the relative abundance for insertional genes, only ISSm2-Xanthob was highly correlated with diaminopyrimidine, glycopeptide, peptide, and tetracycline resistance genes. Meanwhile for integrase genes, only intI1_337old had higher correlations with diaminopyrimidine, glycopeptide, peptide, tetracycline, and fluoroquinolone resistance genes. The plasmid genes (IS26, trb-C) were related with seven different types of ARG. The transposase genes tnpA-02 and tnpA-05 significantly correlated with 10 different types of ARGs (Table S8). The network analysis was conducted to dissect the relationship between individual ARG and MGE (Fig. 5). The result indicated that all the other 15 different MGE genes have significant correlation with different ARG genes, except 5 MGE genes (IS1111, ISPps1-pseud, intl2, pAKD1-IncP-1β, and mobA).

10.1128/msystems.00037-22.9 TABLE S7 Correlation between the relative abundance of MGE and ARG types. Download Table S7, XLS file, 0.02 MB. Copyright © 2022 Wei et al. 2022 Wei et al. https://creativecommons.org/licenses/by/4.0/ This content is distributed under the terms of the Creative Commons Attribution 4.0 International license.

10.1128/msystems.00037-22.10 TABLE S8 Correlation analysis of ARGs and single MGEs (the abundance of ARGs and MGEs is the relative abundance). Download Table S8, XLS file, 0.02 MB. Copyright © 2022 Wei et al. 2022 Wei et al. https://creativecommons.org/licenses/by/4.0/ This content is distributed under the terms of the Creative Commons Attribution 4.0 International license.

### Co-occurrence pattern of ARGs and bacteria genera.

To uncover the co-occurrence pattern of ARG and bacteria, the network analysis was established based on strong and significant correlations among bacterial genera and ARGs (Spearman’s *r* > 0.70, *P < *0.05). The network consists of 131 nodes and 515 edges. As shown in Fig. 5, the co-occurrence analysis results indicated that eight bacterial genera may serve as the potential hosts for different ARG. Among these eight genera, *Psychrobacter* mainly carried the transposase gene (TN5403), aminoglycoside resistance gene [APH(6)-Ic], and MLSB resistance gene (mphA), whereas *Micrococcus* and Staphylococcus were the hosts of MLSB resistance genes (ErmB and ErmQ). *Pelomonas* and *Sediminibacterium* were the potential hosts of aminoglycoside resistance gene [AAC(3)-IV], while *Paracoccus*, *Vagococcus*, and *Jeotgalicoccus* mainly carried the glycopeptide resistance gene (vanHB), multidrug resistance gene (emrD), and other resistance gene (fabK), respectively. These eight bacterial genera mainly can be accurately identified as belonging to the *Proteobacteria*, *Firmicutes*, and *Bacteroidetes* phyla (Table S3). Additionally, the network showed that the potential pathogen *Firmicutes* was a common potential host of ARGs [aadA7, APH(4)-Ib, erm(O), and tetD], transposase gene (tnpA-6), and plasimd (trb-C).

## DISCUSSION

The growing prevalence of AMR is a global concern, and the recognition of the importance of antibiotic resistance is almost pervasive (24), while the role of tick in the dissemination of AMR is unclear. To address this knowledge gap, the microbiomes and resistomes of the wild ticks from 8 different districts in China were explored based on 16s rRNA sequencing and HT-qPCR in this study. According to our results, we concluded that the bacterial communities in wild tick populations from different locations are distinct from each other. Our results indicated that wild ticks are an important reservoir of ARGs, and the antibiotic resistomes of the wild tick population collected from different districts vary significantly in the relative abundance but are similar among them in the number and type. To our knowledge, this is one of the first studies from wild ticks revealing the profile of the microbiome and resistomes.

The abundance of the *Proteobacteria*, *Firmicutes*, *Bacteroidetes*, and *Actinobacteria* phyla accounts for a large proportion of the microbiota among the wild tick samples from different districts, which support findings from earlier tick microbiome studies (25, 26). At the genus level, *Coxiella*, *Staphylococcaceae*_Staphylococcus, *Pelomonas*, *Burkholderia*, and Acinetobacter are the most abundant genera with the averagely relative abundance. Both *Coxiella* and Acinetobacter are potentially opportunistic pathogens (Fig. 2B). The genus *Coxiella* in ticks includes both symbiotic and pathogenic bacteria, and both types in the family Coxiellaceae can cause morbidity and mortality in humans and animals (27). The Acinetobacter was found in a wide range of environments, including soils, water, and air (28). Acinetobacter in wild ticks may have originated from the ecological niches, suggesting that the wild tick microbiota maybe also influenced by bacterial transmission. Six potential pathogens have been detected in wild ticks from different districts. These potential pathogens presented in wild ticks may allow transfer genes to other microbiota making wild ticks a useful reservoir for pathogens and pose high risks to public health.

The wild ticks have been shown in this study to contain 100 ARGs, and these observed resistance genes conferred resistance against 12 classes of antibiotics (Fig. 3). This number is significantly higher than that observed in humans (5 classes) , mouse (4 classes), pigs (5 classes), and soil (5 classes) in previous studies [29, 30], but were slightly higher than those detected in black soldier fly (9 classes) and birds (9 classes) in recent studies (8, 31). The emergence and spread of ARGs are mainly due to the abuse and misuse of antibiotics. Many researches have verified that the direct correlation is present between antibiotic use and the degree of resistance (32, 33). In this study, we found that it was similar in the richness of ARGs among wild ticks from different districts, but the relative abundance was varied greatly (Fig. 4). Our previous study showed that antibiotic use in mouse hosts model changed the gut microbiota of Haemaphysalis longicornis reared in the laboratory (34). Further investigation is needed to examine that the correlation between microbiota and ARGs and antibiotic use in wild ticks. In the co-occurrence network (Fig. 5), *Proteobacteria*, *Firmicutes*, and *Bacteroidetes* were the most prevalent phyla, and these taxa were predicted as possible ARGs hosts. Our results are supported by the fact that mobile ARGs are mainly present in *Proteobacteria*, *Firmicutes*, *Bacteroidetes*, and *Actinobacteria* (35). Studies showed that ARGs frequently switched hosts from *Firmicutes* to the other bacteria (36) and *Firmicutes* might contribute to the metabolism of animal hosts (37, 38). In our study, we found that *Firmicutes* were predominant bacteria at the phylum level and also carried different ARGs and MGEs, including one plasmid (trb-C), one transposase (tnpA-6), and four ARGs [aadA7, APH(4)-Ib, erm(O), and tetD] (Fig. 5). *Firmicutes* may cause ARGs pollution to the vertebrate hosts or environment should be of concern. However, as the co-occurrence patterns revealed in this study were based on correlation analysis, the exact carrying hosts of ARGs need to be verified in further studies.

The microbiota would be selected by various antibiotics and acquired antibiotic resistance capability through HGT or other processes (6). HGT is widely recognized as one of the most important evolutionary forces both in prokaryotes and eukaryotes (39, 40). In addition to transmitting tick-borne disease, wild ticks are of particular concern as they may transmit DNA/hormone to facilitate blood feeding from hosts through HGT (17, 18). MGEs play vital roles in the transfer and dissemination of ARGs in various reservoirs. Twenty MGEs were detected in this study, and a significant correlation existed between all MGEs and all ARGs and also among different individual ARG and MGE (Fig. 5 and Table S7), suggesting that MGEs may also play crucial role on the occurrence and dissemination of ARGs in wild ticks. MGEs contributes to HGT of various ARGs, and ARGs associated with MGEs can frequently switch hosts within bacterial community (6). Given that, monitoring of ARGs and MGEs in wild ticks is urgently needed, especially in the areas where antibiotics are widely used, and ticks and tick-borne diseases are prevalent. To some extent, our results revealed that wild ticks may act as a reservoir of various ARGs and MGEs; further studies are necessary to understand whether and how wild ticks transmit ARGs among different hosts and environment.

## MATERIALS AND METHODS

### Tick samples collection and preparation.

A total of 38 tick pools (80 tick samples/pool) representing 8 populations were collected from 8 different districts during 2018–2021, and the detailed information of sampling is shown in Fig. 1. The sampled ticks of individual pools were washed twice with 75% ethanol, followed by twice washed with sterile water to remove any surface microbes. Each tick pool was homogenized, and the supernatant was collected after centrifugation at 12,000 × *g* for 10 min. The collected supernatant from each tick pool was divided into two; one was processed for 16S rRNA amplicon sequencing, while the other was used HT-qPCR.

### DNA extraction.

The collected supernatant for DNA extraction using the DNA Kit (SBS Genetech Co., Ltd., Beijing, China) according to the manufacturer’s instructions. Extracted genomic DNA was detected and quantified via a NanoDrop ND-1000 spectrophotometer (Thermo Fisher Scientific, Waltham, MA, USA) and agarose gel electrophoresis, respectively, and then stored at −80°C before use. The three resulting extracts from the same sample were composited to get a representative DNA sample for further analysis.

### Microbial community analysis.

Microbial community composition was evaluated for all the samples using 16S rRNA amplicon sequencing. The universal 16S primers (341F/806R) with a sample-specific 12-bp barcode were used to amplify the hypervariable V3 to V4 region of bacterial 16S rRNA genes. PCR was performed using a Bio-Rad thermal cycler Model C1000 (Bio-Rad, Richmond, CA, USA). PCR products were purified with Vazyme VAHTSTM DNA Clean Beads (Vazyme, Nanjing, China) and sequenced using the Illumina MiSeq platform at Shanghai Personal Biotechnology Co., Ltd (Shanghai, China).

Microbiome bioinformatics were performed with QIIME2 2019.4 with slight modification according to the official tutorials (https://view.qiime2.org/) (41). Briefly, raw sequences were quality filtered, denoised, merged, and chimera filtered using the DADA2 plugin with DADA2 pipeline (42). Nonsingleton amplicon sequence variants (ASVs) were aligned with MAFFT (43) and used to construct a phylogeny with fasttree2 (44). Taxonomy was assigned to ASVs classify-sklearn naive Bayes taxonomy classifier against the SILVA Release 132 database (45). On average, we obtained 40,682 nonchimeric reads for each tick pool (Table S1).

10.1128/msystems.00037-22.3 TABLE S1 Information on sequencing depth of 16S rRNA genes. Download Table S1, XLS file, 0.02 MB. Copyright © 2022 Wei et al. 2022 Wei et al. https://creativecommons.org/licenses/by/4.0/ This content is distributed under the terms of the Creative Commons Attribution 4.0 International license.

### HT-qPCR of ARGs.

Only 17 tick pools covering 6 different tick populations were further selected for the HT-qPCR analysis. A total of 384 primer sets were used to interrogate the tick samples DNA. These primer sets targeted resistance genes for all major classes of antibiotics (352 primer sets), mobile genetic elements (MGEs; 24 primer sets), the clinical pathogens (7 primer sets), and the 16S rRNA gene. The detailed information of the primers and target gene annotation are shown in Table S2. All HT-qPCRs were performed using SmartChip real-time PCR system (previously Wafergen, Fremont, CA) as described previously by Guangdong Magigene Biotechnology Co. Ltd (46). In brief, 100 nL qPCRs was dispensed into the SmartChip using SmartChip Multisample Nanodispenser. All the qPCRs were performed three technical replicates. A threshold cycle (*CT*) of 31 was utilized as the detection limit, and the amplification was only regarded positive for further analysis when all three replicates showed positive results. The relative ARG copy number was calculated using the following formula, where *CT* is the threshold cycle of target genes in the 384 primers. The relative genes copy number = 10^[(31−^*^CT^*^)/(10/3)]^, and the relative abundance = target gene copies/16s rRNA gene copies.

10.1128/msystems.00037-22.4 TABLE S2 High-throughput quantitative PCR primer sets in this study. Download Table S2, XLS file, 0.1 MB. Copyright © 2022 Wei et al. 2022 Wei et al. https://creativecommons.org/licenses/by/4.0/ This content is distributed under the terms of the Creative Commons Attribution 4.0 International license.

### Data analysis.

The 16S rRNA sequence data analysis was mainly performed using QIIME2 and R packages (v3.2.0). To visualize the shared and unique ASVs among groups, a Venn diagram was generated in the website of https://www.omicshare.com/tools/Home/Soft/venn according to the previous study (47), while heatmaps were generated using Heml1.0 (48). Correlation analysis was performed using the Spearman correlation coefficient in R packages, and the results were visualized using Gephi platform (49). Common statistics and charts were performed using GraphPad Prism 8 (GraphPad Software, San Jose, CA, USA) and Origin 2019b (OriginLab Corporation, Northampton, MA, USA).

### Data availability.

All the raw sequences have been deposited in the NCBI Sequence Read Archive (SRA) under the accession no. PRJNA791134.
